# Natural Infection of Dairy Cows with Bovine Leukemia Virus Affects Immunoglobulin Levels in Saliva and Serum but Not Milk

**DOI:** 10.3390/pathogens10070907

**Published:** 2021-07-18

**Authors:** Monika Dziuba, Vickie J. Ruggiero, Catherine Wilson, Paul C. Bartlett, Paul M. Coussens

**Affiliations:** 1 College of Veterinary Medicine, Michigan State University, East Lansing, MI 48824, USA; dziubamo@msu.edu; 2 Department of Animal Science, Michigan State University, Anthony Hall, 474 S. Shaw Lane, East Lansing, MI 48824, USA; wilso833@msu.edu (C.W.); coussens@msu.edu (P.M.C.); 3 Department of Large Animal Clinical Sciences, College of Veterinary Medicine, Michigan State University, East Lansing, MI 48824, USA; bartle16@msu.edu

**Keywords:** enzootic bovine leukosis, lymphocytosis, proviral load, immunoglobulin A, immunoglobulin M, antibody

## Abstract

Bovine leukemia virus (BLV) is a retroviral infection that disrupts the immune function of infected animals. It is widespread among U.S. dairy cattle. In this pilot study, the average total IgA and IgM concentrations in milk, saliva, and serum samples from BLV ELISA-positive (ELISA+) dairy cows were compared against samples from BLV ELISA-negative (ELISA−) cows using the Kruskal–Wallis test (with ties). The results from ELISA+ cows were also stratified by lymphocyte count (LC) and proviral load (PVL). In milk and saliva from ELISA+ cows, the average total IgA and IgM concentrations were decreased compared to ELISA− cows, although this was only statistically significant for saliva IgM in cows with low PVL (*p* = 0.0424). Numerically, the average total IgA concentrations were 33.6% lower in milk and 23.7% lower in saliva, and the average total IgM concentrations were 42.4% lower in milk and 15.5% lower in saliva. No significant differences were observed in the total serum IgA concentrations, regardless of PVL and LC. The total serum IgM from ELISA+ cows was significantly decreased (*p* = 0.0223), with the largest decreases occurring in the highest PVL and LC subgroups. This pilot study is a first step in investigating the impact of BLV on mucosal immunity and will require further exploration in each of the various stages of disease progression.

## 1. Introduction

Bovine leukemia virus (BLV) is an oncogenic deltaretrovirus of cattle. Infected animals may develop lymphosarcoma (about 5%) and/or lymphocytosis (about 30%) [1], and many types of immune dysfunction [2,3]. BLV is primarily transmitted horizontally by the transfer of infected cells. In commercial dairy cattle, this is commonly thought to be the result of management practices that result in blood-to-blood contact, direct contact, or by the ingestion of unpasteurized milk [4,5,6]. BLV predominantly infects B-lymphocytes, and the immortalization and clonal expansion [7] of these cells results in the lymphocytosis that develops in approximately one third of infected cattle. Once introduced into a cell, BLV inserts itself into the DNA of that host cell [8], and this proviral DNA can be identified and measured by PCR and qPCR. There is mounting evidence that quantifying BLV proviral DNA (proviral load, PVL) may be useful as an indicator of infectivity and disease progression [9,10,11,12,13], as with the closely related human T-cell lymphotrophic virus (HTLV-1) [14,15,16].

The economic cost of BLV has long been assumed to be limited to morbidity and mortality related to the lymphoma in a small percentage of infected cattle, and lymphomatous tumors remain the leading cause of condemnation for dairy cattle carcasses [17]. However, the economic impact of ‘subclinical’ BLV has been gaining recognition. Recent work has described decreased longevity [18] and milk production [19] in BLV-ELISA-positive (ELISA+) dairy cows as compared to their BLV-ELISA-negative (ELISA−) herdmates. Such findings are in agreement with those of other investigators around the world [20,21,22].

One proposed mechanism for these effects is abnormal immune function in ELISA+ cattle, particularly those with a lymphoproliferative profile (lymphocytosis) [23]. BLV-associated decreases in indicators of systemic immunity (IgG, IgM), altered activation of immune cells, and disrupted T:B cell ratios have been reported in several studies [2,24,25,26]. These immune system abnormalities may explain BLV-associated increases in the incidence of mastitis, hoof problems, and other mucosal diseases [20], as well as failure to clear ringworm infection [24,27]. Despite these reports, the effects of BLV infection on indicators of mucosal immunity are not well characterized.

The aim of this pilot project was to characterize IgA and IgM in dairy cow milk, saliva, and serum associated with BLV ELISA, LC, and PVL. We selected these immunoglobulins because in many mammalian species, IgA is considered the primary immunoglobulin in mucosal immunity and IgM is the primary immunoglobulin in circulation. Although total IgA and IgM concentrations have been described [28,29,30], we found no works to date which have examined the relationship between IgA concentrations and measures of BLV infection. Meiron et al. reported relationships between total IgM concentrations and BLV-ELISA and/or lymphocyte count (LC) status [31], but not PVL. We hypothesized that BLV infection (as determined by ELISA status) would have a negative effect on both circulating and mucosal IgM and IgA.

## 2. Results

Total average IgA and IgM concentrations were determined in milk, saliva, and serum collections from ELISA+ (*n* = 37) and ELISA− (*n* = 19) cows. ELISA+ cows were further subcategorized into specific groups (BLV profiles) based on LC and PVL counts. These subcategories included ELISA+ normal LC (nLC, *n* = 24), ELISA+ high LC (HLC, *n* = 13), ELISA+ low PVL (LPVL, *n* = 25), and ELISA+ high PVL (HPVL, *n* = 9). Comparisons were made between ELISA+ and ELISA− cows, between cows in each ELISA+ subcategory and ELISA− cows, and within ELISA+ subcategories. 

### 2.1. Milk Immunoglobulins

Mean total IgA concentrations in milk are depicted in Table 1. Compared to ELISA− cows, IgA concentrations were 33.6% lower in ELISA+ cows (*p* = 0.3282). This decrease was greatest in ELISA+ cows with nLC and LPVL profiles compared with ELISA− cows, although it did not reach statistical significance. 

Mean total IgM concentrations in milk are depicted in Table 2. Compared to ELISA− cows, IgM concentrations were 42.4% lower in ELISA+ cows (*p* = 0.2991). Following the pattern seen in IgA concentrations in milk, this decrease in IgM concentrations was greatest in ELISA+ cows with nLC and LPVL profiles compared with ELISA− cows, but the decrease was not statistically significant.

### 2.2. Saliva Immunoglobulins

Mean total IgA concentrations in saliva are depicted in Table 3. Compared to ELISA− cows, IgA concentrations were 23.7% lower in ELISA+ cows (*p* = 0.3130). This decrease was greatest in ELISA+ cows with nLC and LPVL profiles compared with ELISA− cows. Within ELISA+ cows, saliva IgA concentrations in nLC cows were significantly lower than HLC cows (57.2%, *p* = 0.0428). Similarly, saliva IgA concentrations in LPVL cows were significantly lower than HPVL cows (66.2%, *p* = 0.0356). 

Mean total IgM concentrations in saliva are depicted in Table 4. Compared to ELISA− cows, IgM concentrations were 15.5% lower in ELISA+ cows (*p* = 0.1729). Following the pattern seen in IgA concentrations in saliva, this decrease in IgM concentrations was greatest in ELISA+ cows with nLC and LPVL profiles compared with ELISA− cows. A decrease of 33.3% in LPVL compared to ELISA− cows was statistically significant (*p* = 0.0424). Within ELISA+ cows, saliva IgM concentrations in LPVL cows were 53.8% lower than HPVL cows (*p* = 0.0224).

### 2.3. Serum Immunoglobulins

Mean total IgA concentrations in serum are depicted in Table 5. Mean IgA concentrations in serum were slightly numerically higher in ELISA+ cows compared to ELISA− cows (*p* = 0.6465). There was minimal difference in mean concentrations between ELISA+ sub-populations of cows. 

Mean total IgM concentrations in serum are depicted in Table 6. Compared to ELISA− cows, mean total serum IgM concentrations were significantly lower in ELISA+ cows (34.3%, *p* = 0.0223). In contrast to the pattern seen in our other results, serum concentrations of IgM in LC sub-categories of ELISA+ compared to ELISA− cows showed a stepwise decrease in mean total IgM: concentrations in nLC cows were 29.2% lower (*p* = 0.0684), whereas concentrations in HLC cows were significantly lower (44.0%, *p* = 0.0273). Compared to ELISA− cows, mean total serum IgM concentrations for LPVL cows were significantly lower (37.4%, *p* = 0.0251).

## 3. Discussion

The objective of this study was to investigate the total IgA and IgM concentrations in the milk, saliva, and serum of BLV ELISA-positive cows with different levels of lymphocytes and proviral load. BLV-associated disruption of antibody levels has been reported in previous studies from our group [2,25,26] and others [24,32]. However, these studies were primarily concerned with serum antibody levels, and did not examine antibody levels in milk or saliva, nor did they assess IgA. We observed a markedly lower concentration of both IgA and IgM in milk and a moderately lower concentration of both IgA and IgM in saliva, although only the decrease in saliva IgM in ELISA+ LPVL cows was significant. Our study also found no statistically significant differences in total IgA concentrations in serum, and our serum total IgM results were consistent with other studies [31]. Within subgroups of ELISA+ cows for both milk and saliva IgA and IgM, this decreased concentration was even more pronounced in ELISA+ nLC or LPVL cows.

Although the total milk IgA concentrations in ELISA+ cows were not statistically significantly different from ELISA− cows, we observed an overall decrease of 33.6% compared to ELISA− cows. The total saliva IgA concentrations were 23.7% lower in ELISA+ cows as compared to ELISA− cows. The total milk and saliva IgM concentrations were similarly decreased in ELISA+ compared to ELISA− cows; we observed 42.4% and 15.5% decreases in milk and saliva IgM concentrations, respectively. These large numerical decreases may have a biological impact that could be investigated in future studies that compare the incidence and severity of diseases mediated by mucosal immunity in subgroups of ELISA+ cows. Our pilot study was small; a larger study size could also provide insight into these declines. The serum IgA concentrations did not differ within ELISA+ cow groups regardless of the sub-category, and we observed a statistically significant decrease in the total serum IgM concentrations in ELISA+ cows compared to ELISA− cows. These results are consistent with other reports of immune disruption in BLV-infected cows. 

Cows categorized as nLC or LPVL had the lowest average IgA and IgM concentrations in both milk and saliva. This result agrees with other investigators who report that BLV infection results in immune disruption even in cows with an aleukemic profile [33,34]. One potential mechanism for BLV-related interference with immunoglobulins is inhibition of the J-chain [35,36], which is essential for the assembly of IgA and IgM polymers as well as for the process of cross-membrane transport [37,38]. BLV is also known to alter a variety of aspects of the immune system that could affect immunoglobulin production, and these alterations often differ based on disease progression. For example, BLV has been shown to disrupt immune signaling cytokines and cause abnormal B-cell responses, and it may dysregulate B-cell apoptosis [23]. The numerically higher concentrations of both IgA and IgM observed in HLC and HPVL cows may be explained by the presence of higher numbers of antibody-producing B-cells in this lymphoproliferative profile. BLV infection may also result in a non-specific activation of B-cells [33], and cytokine disruptions, such as increased IL-2 in persistently HLC cattle, may result in a proinflammatory shift [23] and increased secretion of immunoglobulins from B-cells [39].

Immunoglobulin concentrations in individual cows are highly variable. Reproductive cycles [30], inflammation, and seasonal husbandry [40] have all been reported to influence immunoglobulin levels of many types in individual animals. This pilot study was carried out in naturally infected cows from a single dairy herd where the herd manager was actively participating in a program to reduce BLV by selectively culling cows with HPVL and HLC [41]. Therefore, our study design did not control for the duration of BLV infection, the infecting dose, lactation stage, or individual disease and production histories. Future studies could account for these potential sources of variability by increasing the study population size and utilizing a multivariate statistical model. Cows were selected for this study on the basis of availability, which resulted in low sample numbers, particularly in the HLC and HPVL groups. These disease profiles are associated with immune disruption [24,42]; therefore, the effect of these advanced stages of BLV on antibody levels may have been under-represented in this study in either direction. If additional cows with more progressive disease (i.e., higher LC and/or PVL) were included, we may have seen more extreme increases in antibody levels; alternatively, there may be a point at which more progressive disease results in a “crash” where abnormal immune responses result in a steep drop-off in antibody production. Additionally, previous studies that have demonstrated BLV-associated antibody decreases have focused on neutralizing or antigen-specific antibodies [2,25,26]; however, our study reported no significant differences in total antibody concentrations except in serum and saliva IgM. This may indicate that BLV interferes with antigen-specific immune responses more so than total antibody production, and future studies could be designed to examine antigen-specific IgA and IgM in milk and saliva.

Enrolled cows were sampled as they were available during the production day and were not restricted from food or water. Due to the uncontrollable oral environment, saliva samples were normalized based on total protein concentrations. Saliva proteins, including immunoglobulins, are rapidly degraded in saliva [43]. We attempted to minimize the variability this degradation caused by treating saliva samples with a proteasome inhibitor and storing them at –80 °C; however, our samples were collected from a functioning production facility where immediate freezing and/or centrifugation was not possible. Future studies under more controlled conditions may be able to reduce or eliminate this potential source for variability.

## 4. Materials and Methods

### 4.1. Study Design

Milk, saliva, and serum samples for this study were collected from clinically asymptomatic lactating dairy cows older than two years of age with known BLV ELISA, PVL, and LC results at the Michigan State University (MSU) W. K. Kellogg Biological Station (KBS) Pasture Dairy Center. This herd is a robotic-milking, rotational pasture-grazing/free-stall dairy with approximately 160 cows in the milking herd (~90% Holsteins and 10% Friesians), and was participating in a program to reduce naturally occurring BLV in the herd by selectively culling cows with HPVL and HLC [41]. Based on funding and supply limitations, as well as a limited sampling pool of ELISA+ cows (herd prevalence = 37.4%), 41 cows were randomly selected from the ELISA− and ELISA+ low and high LC and PVL categories. One cow was dry on the sampling day and samples were not collected. Samples of milk, saliva, and whole blood were collected simultaneously from the 40 available cows. Only 5 ELISA− cows were sampled initially; therefore, after securing additional supplies, an additional 16 cows (10 additional ELISA− and 6 additional ELISA+) were selected and sampled in the same manner at the next herd visit approximately six months later (herd prevalence = 27.4%).

### 4.2. Milk, Saliva, and Blood Sample Collection

Milk was manually collected into untreated 50 mL conical tubes and aliquots were frozen at −80 °C after collection. Saliva samples were collected using a SalivaBio Children’s Swab (Salmetrics, Carlsbad, CA, USA) and stored in ice-cooled coolers for transport to the laboratory, where they were centrifuged at 1500× *g* for 15 min, and frozen (at −80 °C). Cows were not restricted from food or water; therefore, to prevent protein degradation due to the uncontrollable oral environment, 50 µg Protease Inhibitor (Pierce, Thermo Fisher Inc., Waltham, MA, USA) was added to saliva samples. Blood samples were collected into a clot activator/polymer gel evacuated tube and a K2 EDTA-treated evacuated tube. Blood in clot tubes was rendered to serum for immunoglobulin ELISA testing. Blood collected into EDTA-treated tubes was separated into aliquots and used for PVL testing and leukocyte counts. Animal procedures for this study were reviewed and approved by the MSU Institutional Animal Care and Use Committee (protocol code 08/16-143-00).

### 4.3. ELISA Test for Immunoglobulins

Commercial antibody ELISA kits (Bethyl Laboratory, Inc., Montgomery, TX, USA) were used to quantify immunoglobulin concentrations following the manufacturer’s instructions. Each assay was optimized to determine the correct dilution factors (Table 7). Saliva samples were normalized based on total protein concentrations following the manufacturer’s protocol using a Bicinchoninic acid assay (BCA; ThermoFisher, Inc., Pittsburg, PA, USA) due to the uncontrollable oral environment. Optical density values were measured at 450 nm using a SpectraMax M5 microplate reader (Molecular Devices, San Jose, CA, USA) and compared to the standard curve run on each plate to determine concentrations. Samples were tested in duplicate on the same plate and the results were averaged; sample pairs were randomly assigned to wells using animal ID numbers.

### 4.4. Lymphocyte Count Determination

Anticoagulated (EDTA) blood samples were collected to measure total and differential leukocyte counts. The first set of samples were analyzed using an automated blood leukocyte differential test (QScout BLD, Advanced Animal Diagnostics, Morrisville, NC, USA) at the MSU BLV Laboratory. This machine was not available at the time of the second sample collection; therefore, blood samples were submitted for leukocyte analysis at the MSU Veterinary Diagnostic Laboratory (Lansing, MI, USA). LC results were categorized as defined in the BLV control program: High (≥7500; HLC), or Normal (<7500/μL; nLC) [41].

### 4.5. Proviral Load Test

The proviral load was measured using the CoCoMo BLV quantitative polymerase chain reaction (qPCR) method [44]. In brief, genomic DNA was extracted from EDTA anticoagulated whole blood samples using the Wizard Genomic DNA Purification Kit (Promega Corporation, Madison, WI, USA). Eluted DNA was normalized to 30 ng/μL, and 150 ng of genomic DNA was added to each qPCR using the BLV-CoCoMo primer set, FAM BLV probe (RIKEN genesis, Tokyo, Japan), and TaqMan Gene Expression Master Mix (Life Technologies, Carson, CA, USA). PVL was categorized as defined in the BLV control program: High (≥50,000; HPVL) or Low (<50,000; LPVL) [41].

### 4.6. Data Analysis

The principal study endpoints were the comparison of total IgA and IgM in milk, saliva, or serum between groups based on (1) ELISA status alone, (2) LC category, and (3) PVL category. Data were assessed for normality by Q-Q plot in Excel (Microsoft, Seattle, WA, USA; raw data) and by kurtosis–skewness and Shapiro–Wilk in Stata 14.2 (StataCorp, College Station, TX, USA; residuals) and were not normally distributed. Between-group differences in total antibody concentrations were evaluated using the Kruskal–Wallis test (with ties) in Stata 14.2. *p*-values of *p* < 0.05 were considered significant.

## 5. Conclusions

In this study, the mean total IgA and IgM concentrations in milk, saliva, and serum were characterized in relation to BLV ELISA, lymphocyte count, and proviral load. The mean total IgA concentrations were decreased in the milk and saliva of ELISA+ cows, but not in the serum. The mean total IgM concentrations were decreased in the milk, saliva, and serum of ELISA+ cows. Although no differences other than the mean total IgM in serum and saliva were statistically significant, these results agree with the growing consensus that BLV infection results in immune system disruptions in cattle. In addition, many diseases of importance in the dairy industry, such as mastitis, are mediated by immune responses at mucosal junctions; therefore, understanding the effect of BLV on IgA could result in improved dairy health. Future research is needed to better characterize the effect of BLV infection on IgA and IgM in saliva and milk, as well as to investigate the potential biological implications of the differences observed in this study.

## Figures and Tables

**Table 1:** Comparisons of average total IgA concentrations in milk.

**Milk IgA Comparisons**						
**BLV** **ELISA Status**	**Avg. Conc. ˆ** **(µg/mL)**	**% Difference**	***p*-Value**	**Median** **(µg/mL)**	**Range** **(µg/mL)**	**N**
ELISA−	62.29	-	-	35.70	1.32–289.36	19
ELISA+	41.37	−33.6	0.3282	28.08	1.19–177.87	37
ELISA+ nLC	40.65	−34.7	0.2214	24.78	1.19–177.87	24
ELISA+ HLC	42.70	−31.0	0.8031	32.58	1.25–129.48	13
ELISA+ LPVL	40.47	−35.0	0.2224	24.74	1.19–177.87	25
ELISA+ HPVL	45.91	−26.3	0.8248	38.67	6.97–114.77	9
**Comparisons within ELISA+ Subgroups**						
**ELISA+** **Subgroup**	**Avg. Conc. ˆ** **(µg/mL)**	**% Difference**	***p*-Value**	**Median** **(µg/mL)**	**Range** **(µg/mL)**	**N**
ELISA+ nLC	40.65	-	-	24.79	1.19–177.87	24
ELISA+ HLC	42.70	+4.8	0.4643	32.58	1.25–129.48	13
ELISA+ LPVL	40.47	-	-	24.74	1.19–177.87	25
ELISA+ HPVL	45.91	+11.9	0.2338	38.67	6.97–114.77	9

ˆ Average Concentration. BLV = bovine leukemia virus; nLC = normal lymphocyte count; HLC = high lymphocyte count; LPVL = low proviral load; HPVL = high proviral load.

**Table 2:** Comparisons of average total IgM concentrations in milk.

**Milk IgM Comparisons**						
**BLV** **ELISA Status**	**Avg. Conc. ˆ** **(µg/mL)**	**% Difference**	***p*-Value**	**Median** **(µg/mL)**	**Range** **(µg/mL)**	**N**
ELISA−	91.07	-	-	53.98	0.60–595.53	19
ELISA+	52.44	−42.4	0.2991	46.38	0.79–167.65	37
ELISA+ nLC	50.25	−44.8	0.2874	46.02	0.79–167.65	24
ELISA+ HLC	56.47	−38.0	0.5267	46.38	19.88–155.36	13
ELISA+ LPVL	48.44	−46.8	0.2654	46.38	0.79–155.36	25
ELISA+ HPVL	60.66	−33.4	0.6055	44.85	21.46–167.65	9
**Comparisons within ELISA+ Subgroups**						
**ELISA+** **Subgroup**	**Avg. Conc. ˆ** **(µg/mL)**	**% Difference**	***p*-Value**	**Median** **(µg/mL)**	**Range** **(µg/mL)**	**N**
ELISA+ nLC	50.25	-	-	46.02	0.79–167.65	24
ELISA+ HLC	56.47	+11.0	0.6332	46.38	19.88–155.36	13
ELISA+ LPVL	48.44	-	-	46.38	0.79–155.36	25
ELISA+ HPVL	60.66	+20.1	0.6819	44.85	21.46–167.65	9

ˆ Average Concentration. BLV = bovine leukemia virus; nLC = normal lymphocyte count; HLC = high lymphocyte count; LPVL = low proviral load; HPVL = high proviral load.

**Table 3:** Comparisons of average total IgA concentrations in saliva.

**Saliva IgA Comparisons**						
**BLV** **ELISA Status**	**Avg. Conc. ˆ** **(µg/mL)**	**% Difference**	***p*-Value**	**Median** **(µg/mL)**	**Range** **(µg/mL)**	**N**
ELISA−	109.79	-	-	42.82	8.00–493.17	19
ELISA+	83.81	−23.7	0.3130	37.96	8.61–597.53	36
ELISA+ nLC	56.49	−48.5	0.0790 *	29.59	8.61–270.93	23
ELISA+ HLC	132.14	+20.4	0.6315	70.37	20.61–597.53	13
ELISA+ LPVL	57.92	−47.3	0.1295	31.92	8.61–270.93	24
ELISA+ HPVL	171.29	+56.0	0.3132	147.87	21.42–597.53	9
**Comparisons within ELISA+ Subgroups**						
**ELISA+** **Subgroup**	**Avg. Conc. ˆ (µg/mL)**	**% Difference**	***p*-Value**	**Median** **(µg/mL)**	**Range** **(µg/mL)**	**N**
ELISA+ nLC	56.49	-	-	29.59	8.61–270.93	24
ELISA+ HLC	132.14	+57.2	0.0428 **	70.37	20.61–597.53	13
ELISA+ LPVL	57.92	-	-	31.92	8.61–270.93	25
ELISA+ HPVL	171.29	+66.2	0.0356 **	147.87	21.42–597.53	9

ˆ Average Concentration; * = *p*-value < 0.10. ** = *p*-value < 0.05. BLV = bovine leukemia virus; nLC = normal lymphocyte count; HLC = high lymphocyte count; LPVL = low proviral load; HPVL = high proviral load.

**Table 4:** Comparisons of average total IgM concentrations in saliva.

**Saliva IgM Comparisons**						
**BLV** **ELISA Status**	**Avg. Conc. ˆ** **(µg/mL)**	**% Difference**	***p*-Value**	**Median** **(µg/mL)**	**Range** **(µg/mL)**	**N**
ELISA−	5.02	-	-	3.99	1.35–12.17	19
ELISA+	4.24	−15.5	0.1729	2.74	0.32–16.93	36
ELISA+ nLC	3.56	−29.1	0.0548 *	2.35	0.32–12.67	23
ELISA+ HLC	5.44	+7.8	0.9694	3.73	1.56–16.93	13
ELISA+ LPVL	3.34	−33.3	0.0424 **	2.41	0.32–12.67	24
ELISA+ HPVL	7.24	+30.8	0.2791	4.22	2.38–16.93	9
**Comparisons within ELISA+ Subgroups**						
**ELISA+** **Subgroup**	**Avg. Conc. ˆ (µg/mL)**	**% Difference**	***p*-Value**	**Median** **(µg/mL)**	**Range** **(µg/mL)**	**N**
ELISA+ nLC	3.56	-	-	2.35	0.32–12.67	24
ELISA+ HLC	5.44	+34.6	0.0809 *	3.73	1.56–16.93	13
ELISA+ LPVL	3.34	-	-	2.41	0.32–12.67	25
ELISA+ HPVL	7.24	+53.8	0.0224 **	4.22	2.38–16.93	9

ˆ Average Concentration; * = *p*-value < 0.10. ** = *p*-value < 0.05. BLV = bovine leukemia virus; nLC = normal lymphocyte count; HLC = high lymphocyte count; LPVL = low proviral load; HPVL = high proviral load.

**Table 5:** Comparisons of average total IgA concentrations in serum.

**Serum IgA Comparisons**						
**BLV** **ELISA Status**	**Avg. Conc. ˆ** **(µg/mL)**	**% Difference**	***p*-Value**	**Median** **(µg/mL)**	**Range** **(µg/mL)**	**N**
ELISA−	342.60	-	-	299.75	178.68–500.27	19
ELISA+	357.19	+4.3	0.6465	362.69	113.45–589.23	37
ELISA+ nLC	348.99	+1.9	0.7692	358.18	113.45–496.34	24
ELISA+ HLC	372.32	+8.7	0.5780	403.03	169.00–589.23	13
ELISA+ LPVL	372.58	+8.7	0.3255	372.45	113.45–586.65	25
ELISA+ HPVL	340.75	−0.5	0.8633	310.75	171.57–589.23	9
**Comparisons within ELISA+ Subgroups**						
**ELISA+** **Subgroup**	**Avg. Conc. ˆ (µg/mL)**	**% Difference**	***p*-Value**	**Median** **(µg/mL)**	**Range** **(µg/mL)**	**N**
ELISA+ nLC	348.99	-	-	358.18	113.45–496.34	24
ELISA+ HLC	372.32	+7.0	0.6792	403.03	169.00–589.23	13
ELISA+ LPVL	372.58	-	-	372.45	113.45–586.65	25
ELISA+ HPVL	340.75	+8.5	0.5714	310.75	171.57–589.23	9

ˆ Avg. Conc. = Average Concentration. BLV = bovine leukemia virus; nLC = normal lymphocyte count; HLC = high lymphocyte count; LPVL = low proviral load; HPVL = high proviral load.

**Table 6:** Comparisons of average total IgM concentrations in serum.

**Serum IgM Comparisons**						
**BLV** **ELISA Status**	**Avg. Conc. ˆ** **(mg/mL)**	**% Difference**	***p*-Value**	**Median (mg/mL)**	**Range (mg/mL)**	**N**
ELISA−	2.07	-	-	1.84	0.45–3.72	19
ELISA+	1.36	−34.3	0.0223 **	1.22	0.06–4.43	37
ELISA+ nLC	1.47	−29.2	0.0684 *	1.29	0.26–4.43	24
ELISA+ HLC	1.17	−44.0	0.0273 **	1.22	0.06–2.29	13
ELISA+ LPVL	1.30	−37.4	0.0251 **	0.99	0.06–4.43	25
ELISA+ HPVL	1.29	−38.1	0.0650 *	1.24	0.64–2.11	9
**Comparisons within ELISA+ Subgroups**						
**ELISA+** **Subgroup**	**Avg. Conc. ˆ (mg/mL)**	**% Difference**	***p*-Value**	**Median (mg/mL)**	**Range (mg/mL)**	**N**
ELISA+ nLC	1.47	-	-	1.29	0.26–4.43	24
ELISA+ HLC	1.17	−25.0	0.4643	1.22	0.06–2.29	13
ELISA+ LPVL	1.30	-	-	0.99	0.06–4.43	25
ELISA+ HPVL	1.29	00.01	0.5847	1.24	0.64–2.11	9

ˆ Avg. Conc. = Average Concentration. * = *p*-value < 0.10. ** = *p*-value < 0.05. BLV = bovine leukemia virus; nLC = normal lymphocyte count; HLC = high lymphocyte count; LPVL = low proviral load; HPVL = high proviral load.

**Table 7:** Optimized dilution factors by antibody and sample type.

Antibody	Sample Type		
	**Milk**	**Saliva (Normalized)**	**Serum**
**IgA**	1:250	1:10,000	1:640
**IgM**	1:400	1:4200	1:10,000 ^1^

^1^ The dilution factor for serum IgM was previously optimized for this assay by Frie et al. [2].

## Data Availability

The data presented in this study are available on request from the corresponding author.
